# Corruption and Population Health in the European Union Countries—An Institutionalist Perspective

**DOI:** 10.3390/ijerph19095110

**Published:** 2022-04-22

**Authors:** Oana-Ramona Socoliuc (Guriță), Nicoleta Sîrghi, Dănuţ-Vasile Jemna, Mihaela David

**Affiliations:** 1 Department of Economics and International Relations, Faculty of Economics and Business Administration, Alexandru Ioan Cuza University of Iași, 700505 Iași, Romania; 2 Department of Economics and Economic Modelling, Faculty of Economics and Business Administration, West University of Timisoara, 16 Pestalozzi Street, 300115 Timișoara, Romania; nicoleta.sirghi@e-uvt.ro; 3 Department of Accounting Business Information Systems and Statistics, Faculty of Economics and Business Administration, Alexandru Ioan Cuza University of Iași, 700505 Iași, Romania; danut.jemna@uaic.ro; 4 Economic Research Department, “Gh. Zane” Institute for Economic and Social Research, Romanian Academy—Iași Branch, 700481 Iași, Romania; mihaela_david88@yahoo.com

**Keywords:** corruption, population health, life expectancy at birth, child mortality, inclusive institutions, extractive institutions

## Abstract

Even though the European Union (EU) is considered one of the best performers in the world in fighting corruption, the situation changes when the analysis is shifted to the national dimension of its member states, with significant differences concerning the effects of corruption on population health. Using the theory of New Institutional Economics as a complementary tool that provides additional representativeness to this phenomenon, the aim of this paper is to empirically investigate the impact of corruption on population health, considering also other demographic and socio-economic determinants. Using data collected at the EU level registered between 2000–2019, we employ panel date models to validate the ongoing effect of perceived corruption on population health. Our empirical findings fully validate the institutionalist perspective, according to which countries with inclusive institutions better control the anomaly of corruption while benefitting from higher life expectancy and reducing child mortality rates. Conversely, the EU countries with rather extractive institutions suffer in terms of both longevity of population and infant mortality. Our study emphasizes that in tackling corruption pressure on population health, the most effective way is to improve the quality of governance in countries with fragile institutions.

## 1. Introduction

Corruption is a real problem for modern society, having serious implications at the social, political, and economic level, while boosting poverty and inequality and weakening public services, investments, and justice. It undermines democracy and the quality of institutions [1]. Corruption vitiates the logic of economic development by encouraging fraud, bribery, and embezzlement, by diminishing tax revenues, boosting shadow economies, and promoting high expenditures with “white elephant” projects which are socially ineffective [2]. Population health is one of the victims of corruption and its inherent misallocation of public resources. People (especially the poor) are deprived of access to basic medical facilities and receive low-quality medical services, so their overall quality of life is harshly affected.

Corruption is a widespread disease that has deep roots in society’s social and political structures [3]. Such “abuse of entrusted power for private gains” [4] is the outcome of a critical situation in which civilizations are lacking sound values such as morality, trust, and integrity, allowing extractive institutions to operate and explaining why nations fail [1,5]. This is also the case for the European Union (EU), where corruption is present at different intensity levels. After the enlargement process, the EU (with multiple development speeds) is providing fertile ground for discrepancies between inclusive or extractive institutions and, consequently, for lower or higher incentives for corruption [1,6]. Corruption is becoming a threat for health conditions, equity, and education, promoting inequalities, poverty, and even mortality [7]. It erodes population health status and, therefore, social welfare. Perceived from a micro-level perspective, corruption affects households, firms, and people [8,9]. From a macro-level point of view, it determines insufficient budgetary funds for the health sector and lower quality provision of health services at higher costs [10].

Within the EU, some countries are experiencing serious population health problems because of corruption. On the one hand, the majority of Western and Northern economies have a tradition in keeping corruption under control through effective regulations and higher transparency in proving financial support to the health coverage of the entire population. Consequently, their health outcomes are less harmed. On the other hand, other European countries, especially the Central and Eastern countries, are more exposed to such phenomenon due to their milder rules concerning corrupt behaviors. This provides fertile ground for weakened health conditions. In other words, the former group of EU countries are benefiting from a solid institutional basis (inclusive institutions, in the terms of Acemoglu and Robinson [1]) or good economic, political, and legal rules that protect individuals and their basic needs, including health. Conversely, the latter group of countries have a rather poor institutional basis (rather extractive regulations), mainly for nations which have experienced a communist regime in the past and are more exposed to a higher degree of corruption, and thus, to inequality and fragile health conditions. As an economic perspective, New Institutional Economics provides depth to our argument because it allows analysis through time and space and traces the origins of the evolution or, conversely, the involution of a country through its past historical, cultural, and political experiences, and also through the existing formal and informal rules which shape human interaction. If formal regulations express human intentionality, being created by people who have the power to impose them, then informal institutions highlight values, traditions, and mindsets naturally modelled over time, having a strong inertial character.

Differences of corruption intensity among the EU can also justify the failure of its member countries in accomplishing Millennium Development Goals (MDGs), with precise targets for poverty, hunger, and child mortality. Corruption denies health and hunger [10]. It negatively influences the quality of health professionals, medical personnel, medical research and innovation, quality of medical equipment and access to it, and the supply of medicine and provision of healthcare facilities in rural and urban areas [7,11]. Corruption also has a negative impact on healthcare satisfaction [8,12]. As expected, corruption’s effects are harsher in developing countries than in developed ones [13]. In 2016, Romania, for example, was far behind most EU countries, with the highest level of child mortality from the EU (141% higher than the EU average) and serious troubles with corruption, having a Corruption Perception Index (CPI) rating under the global average, at 48 points out of 100 [10,14].

Our research intends to fill the existing gap concerning the nexus between corruption and population health from the perspective of an outside approach to the topic. To the best of our knowledge, previous papers have mainly considered the econometrically validated impact of corruption on population health without going further and trying to perceive corruption as an institutional anomaly whose influence on health is currently in progress. Therefore, the aim of this paper is to investigate how corruption, as an outcome of extractive institutions, is affecting population health at the level of the EU. Using the perspective provided by New Institutional Economics, our paper intends to go further and trace the origins and dynamics of corruption in the social, political, and economic climate of the analyzed sample of countries. Thus, considering the EU, we emphasize that health issues are more prominent where corruption, as consequence of ineffective regulations, is high and less problematic in those countries where corruption pressure is limited due to its inclusive institutions. Our endeavor will provide added value to the existing body of literature from at least two perspectives. First, by using the theoretical background of New Institutional Economics, via Acemoglu and Robinson [1], we explain why some EU nations have failed in keeping corruption under control and harmed population health in the long run, while others succeeded at creating auspicious conditions for positive health outcomes. Second, we have focused our analysis exclusively on the case of EU countries in a selected time span between 2000–2019. Using variables such as total life expectancy at birth, child mortality (under 5 years), rank in the Corruption Perception Index, GDP per capita, unemployment rate, age dependency ratio, general government education expenditure (as % of GDP), general government health expenditure (as % of GDP), and degree of urbanization, we show that better health outcomes are associated with lower corruption, higher economic development, increased attention paid to education expenditures, higher levels of urbanization, and a lower age dependency ratio. Employing panel data models, we show that the nexus between corruption and population health fully validates the theory of New Institutional Economics, which should be further perceived as a complementary tool that provides additional representativeness to the phenomenon.

The remainder of the paper is structured as follows. Section 2 reveals the most important findings from the vast body of literature on the relationship between corruption and its impact on population health within EU countries, with a particular focus on the institutional roots of corruption. Section 3 presents the data and methodology employed in this study. Section 4 emphasizes the main empirical results. Section 5 presents the discussions and Section 6 concludes.

## 2. Literature Review

In our endeavor to explore the literature on the topic of corruption and its impact on health outcomes, we have analyzed previous research from the perspective of analyzing existing shortcomings. On the one hand, we have found that little attention has been paid to the reasons for why nations fail in diminishing corruption and at improving the health status of their citizens, and, moreover, that the corruption–health nexus is not perceived as an ongoing process. To the best of our knowledge, no theoretical background provided by New Institutional Economics has been employed in the literature, where institutions (formal or informal, and inclusive or extractive) are responsible for the progress or, conversely, for the regress of a society in all its dimensions, including health. On the other hand, few papers dealt with the relationship between corruption and health outcomes at the EU level. Consequently, we do believe that institutionalist theory should be fruitfully explored so as to provide depth to our argument. Institutions matter for development. Therefore, their quality and effectiveness are directly responsible for the outcomes generated for population health. When institutions are not effective, corruption flourishes and undermines development while vitiating population health.

### 2.1. Why Institutions Matter for Population Health?

Institutions are the ‘rules of the game’, or constraints created by people to shape human interaction. On the one hand, they can be formal (official), representing written economic, political, and social rules that are the result of human intentionality and are more flexible, admitting subsequent adjustments in accordance with the needs of society. On the other hand, institutions can be informal (unofficial) or unwritten norms, reflecting values, traditions, norms of conduct, and codes of behavior that come from the past and have the power to determine the evolution of a society [15,16,17]. Unlike formal regulations, informal rules are rigid and need long periods of time to change; decades, or even centuries [18]. Acemoglu and Robinson [1] go further and explain why nations fail at creating prosperity on the basis of institutional effectiveness, and they refer to good governance as inclusive institutions (political and economic rules created in order to defend society and improve living conditions) and bad governance as extractive institutions (political and economic norms created in order to satisfy the needs of those who have the power, accompanied by the use of coercion, limited freedom, and poor quality of life for the rest of the people). Consequently, the quality of these rules is heavily influencing the social and economic dynamics of countries towards a positive or negative path. One significant outcome is reflected in the health of the population. Good (inclusive) institutions create auspicious conditions for civilization, human rights, education, health, freedom, democracy, innovation, trade, and progress. Bad (extractive) institutions are more often associated with dictatorship, obedience, hunger, poverty, and even mortality. Political factors have a determining role in this respect because political institutions are the ones which shape further economic regulations. Consequently, their quality is highly dependent on the political orientation of those who propose them.

In the extension of such a perspective, health should be one of the basic directions targeted by the structural policies of the state. Governments that promote clear rules to protect their citizens while ensuring the population (mainly the poor) has access to better healthcare services will discourage acts of corruption. This can be explained in light of two stances. First, a healthier population with a higher life expectancy is a basic condition for higher labor productivity, where the increased physical and mental capacities of workers will positively influence the labor market, decreasing the pressure of nepotism and unemployment [19]. Second, healthy children have a higher capacity to learn and further expand their cognitive development, an aspect that fosters school attendance and shapes more educated future generations; a necessary precondition for a less-corrupt society [20]. In a nutshell, health is a barometer of the political and socio-economic conditions of a country, capturing the level of institutional effectiveness. From a micro-level perspective, population health is highly determined by specific inner characteristics of the people, such as genetic profile, age, gender, and race [21]. From these basic traits, people embrace habits and routines, which are inspired and designed in accordance with the environment they live in. Practically, individuals are circumscribed to a social network (family, friends, and local community), adopting attitudes and behaviors, moral values, codes of conduct, and norms of behavior which are dominant and representative for that community [15]. All these are generically called informal institutions, which come from the past and are perpetuated from one generation to another through imitation and learning processes. In other terms, people acquire a sort of inertial routine and become part of the macro-level perspective [22,23,24]. From this latter point of view, health depends on a broader set of social, working, and living conditions, meaning education, income, occupational status, quality of life, routines, social rules and norms, and even environmental conditions [25]. However, their quality is the result of the effectiveness of formal institutions (the economic, legal, and political ‘rules of the game’) [17,26,27,28,29].

Good governance (democracy as a meta-institution, strong rule of law, trust, and civilization) is therefore a precondition for people’s wellbeing, especially when dealing with the health dimension [30,31]. According to Aisa et al. [32], when governments are effective and boost expenditures for public health, a long-term positive outcome will appear. A large mass of the population will benefit from access to health services and, thus, longevity will increase. Moreover, other papers highlight that higher public support for providing healthcare services is correlated with a sharp decrease of infant mortality under 5 years and child mortality in general [33,34,35]. Government effectiveness is a barometer of institutional quality and has a large influence on health status via the instrument of governmental spending on the health sector and the protection of civil rights by proper regulation. When preventative healthcare services are supported by public money, they are more likely to support individuals with limited financial possibilities, who otherwise could not benefit from such care [33]. Consequently, more lives are saved, the workforce is more productive, and people do not need to appeal to corrupt practices to benefit from their rights [36]. Democracy, political rights, and civil liberties are the institutional underpinnings of good population health [37].

The exertion of power for personal purposes highlights abuses which take place first at the political level, meaning that political institutions are the prime source of corruption [2,4,28]. If political institutions are vitiated, obviously, all the economic and social regulations that the political class shape will already be projected towards a negative path, encouraging the misallocation of resources, corruption, and loss of tax revenue, retarding thus the economic progress [2]. The EU is experiencing different levels of corruption which generate disproportionate damaging effects on health systems. Problems are more prominent in countries with harmed economic and political institutions, such as former communist countries, than in Western nations [3,38]. In the literature, corruption is measured by several variables, such as CPI (in most cases), control of corruption as a component of the Worldwide Governance Indicators, the PRS Group’s Corruption Index, and the ICRG’s corruption level, and there is a unanimous perspective concerning the negative impact of corruption on health [8,39,40,41].

**Figure 1:**
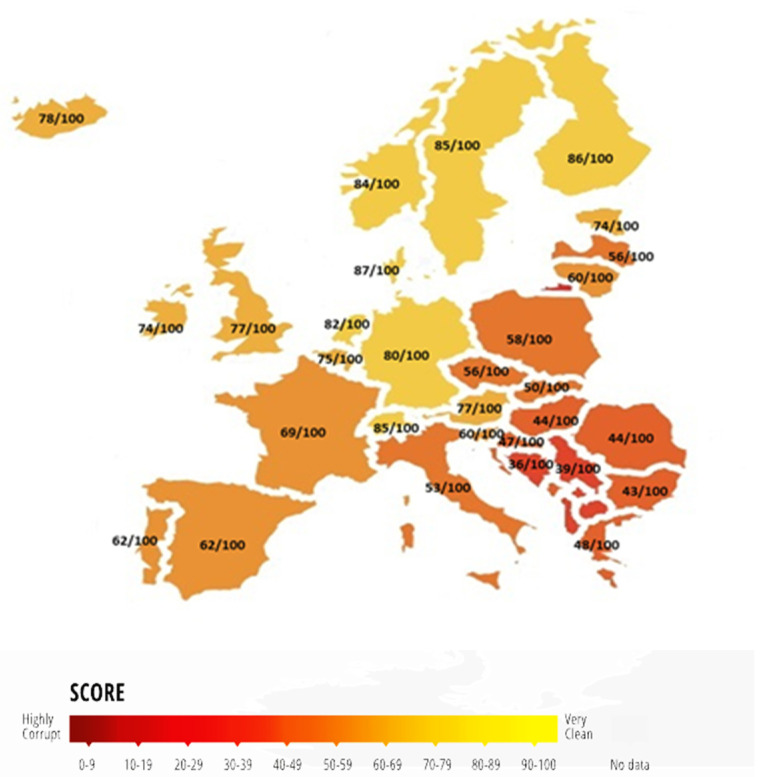
Corruption Perception Index among EU countries in 2019. Source: Authors’ representation using data from Transparency International, 2019.

EU member states are committed to the core EU values, such as human dignity, freedom, democracy, equality, rule of law, and human rights [42], but the effectiveness of the institutional background and freedom from corruption have a rather national dimension. The major divergence between the Western and the Eastern EU countries resides in the fact that the Western countries have the full benefit of democracy, freedom, rule of law, and political rights in building inclusive institutions that protect its citizens from abuses. In some Eastern nations, centralized planning, coercion, obedience, forced collectivization, and mass destruction of private property has harmed their institutional background, creating poor and ineffective norms even after the fall of communism [15,17]. Practically, all countries highly depend on their past, some in a good way and others in a bad way. Consequently, even though the EU is the best performer in the world at reducing corruption, according to the rankings of the CPI for 2019 [43], the situation is totally different when the analysis is shifted to the country level. In 2019, the EU had Denmark, Finland, and Sweden as its best performing countries with the highest rankings in the CPI, indicating low corruption, but also Bulgaria, Romania, and Hungary in the opposite situation, with the highest corruption levels [44]. A more detailed image of perception towards corruption among all EU countries for 2019 can be observed in Figure 1 below, where we can see the full rankings of member states.

Obviously, the negative effects of corruption on health are more pronounced in developing economies because these emerging countries have weaker institutions vitiated by the communist experience. The CPI has become the leading global index of public sector corruption [4] and is used systematically in studies that look at the impact of corruption on population health and health systems’ performance, assuming the limitations of this measure [1,8,39,40,41,45,46,47,48,49]. Even though we deal with a subjective measure of corruption, the index is complex and the data used to develop it refer to several ways in which this phenomenon manifests itself: bribery, diversion of public funds, nepotism, legal protection, access to information for officials, etc. [50]. There are studies using other measures of corruption, such as control of corruption, provided by the World Bank’s Worldwide Governance Indicators [51,52], but for our sample of countries we did not have available data for the whole period. However, this index also measures perceptions of corruption, and a comparative analysis of the advantages and limitations of the two indicators is a topic of discussion in the literature [53]. Therefore, problems such as the diversion of public funds, officials’ abuse of power for private gain, nepotism, bribery, excessive bureaucratic burden, legal ineffectiveness, and a lack of transparency with respect to public affairs appear mainly within societies where both informal and formal institutions create auspicious conditions. Hence, good governance (inclusive institutions) is highly correlated with higher life expectancy and a lower rate of infant mortality [45], while poor governance (extractive institutions) is reflected in the opposite perspective, harming population health [1,54].

### 2.2. The Curse of Bad Institutions on Population Health–Corruption’s Effects on Life Expectancy and Child Mortality

The nexus between corruption and population health is mostly explored within papers based on macro data with longitudinal series that use variables such as life expectancy and infant mortality as proxies for health. Life expectancy highlights a projection of how long people are expected to live, assuming the fact that mortality rates by age remain constant. Obviously, such an indicator has a national dimension. It provides significant information concerning the access of a population to healthcare services and their quality, or about the level of expenditure and financing dedicated to support health [54]. Life expectancy at birth is also a reflection of local conditions, with respect to the demographic, economic, and social context (income, education, living conditions, institutions, etc.). Consequently, in developing countries, where conditions are poor and the imminence of corruption is higher, life expectancy is decreasing [39]. There is a generous body of literature focused on the topic of corruption and life expectancy highlighting that corruption has a major impact on access to health facilities and the quality of health services, and these, in turn, further affect life expectancy at birth of the population. Gaitonde et al. [55] and Nadpara et al. [13] emphasize that corruption reduces the effectiveness, efficiency, and equity of health services, harming life expectancy and, thus, population health overall. Using cross-sectional data of more than 120 countries, Holmberg and Rothstein [45] pointed out that countries with effective or inclusive institutions (strong rule of law, low levels of CPI, and increased government effectiveness–variables which they present as indicative of the quality of government) have higher levels of life expectancy. The EU nations that have a higher life expectancy are less open to corruption, because people are well educated, live in better conditions, and have better access to healthcare services. Those EU countries with lower life expectancy have the features of insufficient finance allocated to the health sector, low quality of life, limited education, and inefficient rules that open the road towards corruption. Such inauspicious context is diminishing people’s longevity and, even more, patients’ safety [40,46]. Therefore, where corruption is high, the quality and equity of health services decrease, and, unfortunately, from all sectors, health tends to be particularly more exposed to deviant practices [47,48]. People with poor health conditions will be more willing to offer bribes to benefit from various healthcare services because, otherwise, they will be left outside the system [56].

Another important variable that captures health status is infant mortality, one of the top priorities of the Millennium Development Goals, the trend of which is expected to sharply decrease until 2030. Corruption determines life losses, especially of children, through the lack of expenditure made by the government to support the healthcare sector. Corrupt governments are responsible for increasing the number of poor people that depend on services provided by them, such as healthcare, and make the population more exposed to bribes when they need access to healthcare services. Holmberg et al. [45] show that mortality rates are higher where corruption is more prominent. Studies have pointed out not only a long-term positive correlation between corruption and infant mortality [8,57,58] but also a bidirectional relationship between these two variables [57]. On the one hand, high corruption exposes children to death because of the unavailability of health services which are supposed to save lives. On the other hand, poor people that are at risk of being left outside the health system are the ones who need to make informal payments to obtain access to it, stimulating corruption. In their endeavors to estimate the cointegrating relationship between corruption and infant mortality in Turkey, based on a self-constructed index of corruption ranked between 1960 and 2010, Dincer et al. [58] emphasize that corruption indirectly stimulates infant mortality through income inequality. People with low income will have to pay a larger share of their personal budget as bribes; consequently, those that are unable to offer bribes are more exposed to death than the rest. Because of precarious economic growth, vulnerable or poor societies deal with a higher risk of infant mortality. The reason for this is that corruption also affects economic progress by impeding developing economies in their efforts to catch up to developed economies [2,59]. Consequently, corruption could be an indirect source of more than 140,000 annual child deaths worldwide [60].

There are also other important factors related to demographic, social, and economic context which are taken into consideration when the issue of population health is addressed. Various studies have found evidence for a significant association between economic performance and population health. More specifically, Achim et al. [39] found that a strong economic strategy that would increase GDP per capita reduces the mortality rate for children under 5 and increases life expectancy. These results are consistent with many other studies in the field highlighting that higher levels of economic development are linked to better health outcomes [61,62,63]. Moreover, Hanf et al. [11], Gordon and Biciunainte [64], and Li et al. [51] support the idea that a higher income provides better access to housing, education, and health services which lead towards larger improvements to health, lower mortality rates, and longer life expectancies. Previous literature also emphasizes the substantial effect of public health spending on health indicators. In this respect, some scholars find empirical evidence for the negative correlation between the mortality rate of infants and children and the share of health expenditure as a percentage of Gross Domestic Product (GDP), or sanitation [11,65,66,67], while others prove that higher public health expenditures have a significant contribution in increasing life expectancy at birth [51,54]. Nevertheless, according to Rajkumar and Swaroop [68] and Factor and Kang [69], the impact of public health expenditure on health outcomes is often insignificant, especially in poorly governed countries. Among other broader socio-economic and demographic factors with significant beneficial impacts on population health is education. For example, public spending on education has a significant impact on reducing infant mortality [58], while better education coverage over time is associated with substantial gain in life expectancy [54,70,71]. Far fewer studies include unemployment as a health determinant, and when using macro-level data, mixed results are found [54,72,73]. Conversely, studies using micro-level data find more consistent results which sustain that unemployment adversely affects both mental and physical health [74,75] and is associated with higher risk of mortality [76]. In accordance with Li et al. [51], another important determinant of population health is urbanization. In this regard, the authors stress that increased urbanization decreases mortality rates and increases life expectancy. Another range of studies focus on analyzing the health indicators in relation to population aging, underlining the direct implications of this phenomenon on the degree of social and economic dependency of the elderly population and the pressure on the pension and social security system, which may further lead to a negative impact on health status [77,78].

## 3. Materials and Methods

We aim to empirically assess the effects of corruption at the institutional level on population health in EU countries. We begin by presenting the data, and then we expose step-by-step our econometric strategy.

### 3.1. Data—Representative Sample and Measurement Units

We use data for all 28 EU countries (Appendix A) over the period 2000–2019. Except for CPI, which is provided by Transparency International, the rest of the data are collected from the World Bank and EUROSTAT, as presented in Table 1 below.

To measure population health, we refer to total life expectancy at birth and child mortality rate under 5 (per 1000 live births). The main independent variable is the CPI, which is used to measure the perceived levels of public sector corruption in EU countries. If until 2011, the methodology for assessing corruption of countries all over the world involved calculating an index with values ranging from 0 (highly corrupt) to 10 (corruption free/very clean); since 2012, there has been a change in this methodology which has also led to a change in the measurement scale, with CPI taking values from 0 (highly corrupt) to 100 (corruption free/very clean). Therefore, in accordance with Achim et al. [39], in our study we use ranks from 1 (lowest level of corruption) to 28 (highest level of corruption), determined based on the index values assigned to the EU countries. The two main proxies for population health are also regressed, besides the rank of corruption, to a set of socio-economic and demographic determinants using the literature: Gross Domestic Product (GDP) per capita (current US $); unemployment rate; age dependency ratio (% working-age population); general government education expenditure (% of GDP); general government health expenditure (% of GDP); and degree of urbanization. The descriptive data on the variables are provided in Table 2 as follows.

### 3.2. Research Methodology—Models of Analysis

For estimating the impact of corruption on population health, our study relies on a panel data model over the 2000–2019 period. The reason for choosing this time span resides in the limited availability of data for each country, which allowed us to work with a balanced panel.

The first step in our econometric strategy when working with panel data is to test the stationarity of the time series, both at the level of each country and at the level of the whole panel. Taking into consideration that the time dimension, as well as the cross-section dimension, are relatively small, the literature suggests using three types of panel unit root tests. On the one hand, the first perspective, developed by Levin, Lin, and Chu [79], assumes the null hypothesis of a common unit root for each panel against the alternative that all panel data series are stationary. On the other hand, the Augmented Dickey Fuller (ADF) Fisher-type test proposed by Choi [80] and the one developed by Im, Peseran and Shin [81] test the null hypothesis, according to which all panels contain a unit root against the alternative of at least one stationary panel.

The baseline model specification for estimating the effect of corruption on health condition of population is:

(1) $$Health_{it}=\beta_{0}+\beta_{1}Rank\_CPI_{it}+\beta_{2}X_{it}+\mu_{i}+\lambda_{t}+\varepsilon_{it}$$

where $Health_{it}$ indicates health outcomes in each 28 EU countries i and over 20 years t, alternatively including total life expectancy at birth and under-5 child mortality rate, $Rank\_CPI_{it}$ is the main independent variables illustrating the rank of each country i according to CPI, $X_{it}$ includes a set of other independent variables, $\mu_{i}$ is the unobserved country-specific fixed effects which account for differences across countries that are time-invariant, $\lambda_{t}$ is the year-specific fixed effects which control for factors that vary uniformly across countries, and $\varepsilon_{it}$ is the error term assumed with conditional mean zero and independent of $RankCPI_{it}$ and $X_{it}$.

When applying the fixed effects estimator, it is important to remember that while heteroskedasticity in the residuals is always a potential problem, nothing rules out serial correlation [82]. Therefore, we use the White [83] robust standard errors test to address these problems. The validity of fixed effects model is tested by performing an F-test, with the restricted residual sums of squares (RRSS) being that of OLS on the pooled model and the unrestricted residual sums of squares (URSS) being that of the fixed effects regression [84]. A significant F-test indicates that including country- and time-specific effects is preferable to a simple OLS regression.

As robustness checks, a random effects estimator is also used, which, besides controlling for unobserved heterogeneity, can also account for variation both within and between countries. In order to test the difference between the fixed and random effects estimators, the specification test proposed by Hausman [85] is used. If the null hypothesis is rejected, the random effects estimator is not consistent.

Finally, the System GMM estimator is also used as a second robustness check, since, besides controlling for unobserved heterogeneity and non-stationarity, it can also deal with the potentially endogeneity of explanatory variables and measurement errors, as well as any potential autocorrelation and heteroscedasticity in the panel [86,87].

## 4. Results

This section presents the main modelling results on the impact of corruption and other socio-economic and demographic determinants on population health, following the previously discussed steps regarding the methodology for panel data.

### 4.1. Stationarity

To assess the stationarity of all variables considered in the analysis, we draw upon the LLC, IPS, and Fisher-ADF unit root tests, the results of which are provided in Table 3. Both the trend and the intercept are included in the autoregressive specification of each test to verify for both difference and trend stationarity.

As Table 3 illustrates, the rank of corruption, unemployment rate, age dependency ratio, and general government education expenditure are stationary since, irrespective of the test, the null hypothesis of the presence of a unit root is rejected. Instead, all the other variables, i.e., total life expectancy at birth, child mortality rate, GDP per capita, general government health expenditure, and urbanization, are non-stationary as the combined results from all these tests indicate the presence of a unit root. Moreover, as the low *p*-values point out, these variables are stationary in the first difference, again regardless of the considered test.

### 4.2. Findings

The results of estimating the impact of corruption on population health, controlled for other socio-economic and demographic determinants, are provided in Table 4 and Table 5: Table 4 reports the results for panel data models capturing the influence of these factors on total life expectancy, and Table 5 illustrates the response of the second measure of population health, i.e., infant mortality under 5, to changes in these factors over time and across countries. In order to control for any unobserved country-specific characteristics or any unobserved time shocks, the baseline panel data model includes both country- and time-fixed effects. The findings corresponding to these models are displayed in column 1 in Table 4 and Table 5. For both models, the F-test supports the significance of fixed effects estimates, indicating that including country- and time-specific effects is preferable to a pooled OLS regression. To further demonstrate the validity and the robustness of the findings, we consider the alternative regression model with joint country- and time-specific random effects (the results are provided in column 3 in Table 4 and Table 5). Even if the results of these two regression methods are qualitatively similar, models that include fixed effects are indicated as preferable by the higher goodness of fit, as well as by the Hausman test. Finally, endogeneity may appear due to possible reverse causality between variables. For example, the bidirectional relationship between corruption and population health may be explained by the fact that corruption is jointly determined with the access of people to healthcare services [57]. Moreover, the other independent variables such as GDP per capita, unemployment, age dependency ratio, and degree of urbanization can be also endogenous because they reflect the institutional quality at the national level of the EU member countries. Considering that potential bias in these estimates may be caused by the endogeneity of explanatory variables, further on we use the GMM estimation system to address these problems (the results are reported in column 4 in Table 4 and Table 5). Therefore, we consider the latter specification to be preferable and comment the results accordingly.

Overall, the estimation results reported in Table 4 reveal that corruption has a negative impact on total life expectancy at country level in the EU during the sample period (Model 1), although the inclusion of country- and time-specific random effects makes the result statistically insignificant (Model 2). However, when considering the endogeneity problem (Model 3), the result is robust and validates the negative response of life expectancy to corruption. These findings are in line with existing studies conducted both at the European [40,46,47,48] and global level [39,45,51] and highlight that a lower level of corruption is related to better health outcomes, measured by total life expectancy. When looking at the economic performance of EU countries, our results reveal that GDP per capita has a significant contribution to increasing total life expectancy. In other words, we can conclude that people living in countries with a higher level of economic development can, on average, expect to live longer in comparison to those residing in countries with lower economic performance. These results are in accordance with other empirical studies [39,51,64,88] emphasizing that higher levels of economic development impact all sectors of a society—by improving the access to education, health services, social services, etc.—and thus enhance the people’s quality of life, which obviously tends to increase their life expectancy. Nevertheless, as Achim et al. [39] point out, the association between GDP per capita and life expectancy tends to be weak after reaching a certain threshold. Turning to the unemployment effect upon life expectancy, the result implies that, when the possible endogeneity is handled, the impact of unemployment on life expectancy is no longer significant. Our empirical findings are consistent with the results obtained in the literature [54], which highlight that unemployment could not be nominated as a driven factor for life expectancy. Unlike unemployment, age dependency ratio shows a significant negative influence on life expectancy in all three models (Table 4). These empirical findings suggest that those countries with their young and elderly population more dependent on the working-age population have lower levels of life expectancy. The main explanation for such an outcome refers specifically to the pressure on the public pension and health insurance systems to address the specific needs of the elderly population [77,78], but also to the focus of governments on improving access to quality education, learning opportunities, and healthcare for children and adolescents throughout their lives [89]. Inequalities related to these vital spheres of society, both between and within countries, are further deepened by low levels of urbanization. Our results are consistent with the existing literature, sustaining that a higher level of urbanization leads to an increase in life expectancy because the urban population has higher access to education, healthcare services, the labor market, and higher incomes [90]. Government education expenditure (as a proxy for accessibility to education) is also positively associated with life expectancy, supported by extensive literature [71]. However, when looking at accessibility to healthcare services, our findings reveal that higher government expenditure has no significant impact on life expectancy in models 1, 2 or 3. These findings are consistent with Factor and Kang [69], who suggest that health expenditures are not directly associated with health outcomes. Particularly for the sample period and at the level of EU countries, health expenditure as a percentage of GDP has maintained its upward trend with more or less marked fluctuations for most countries, with the exception of Hungary, the only country to show a significant decrease in health expenditure since 2003, and Ireland, Portugal, Greece, and, to some extent, Lithuania, for which a downward trend started in 2009—which is obviously one of the effects of the austerity measures pursued during the Great Recession [90]. For EU countries during the selected time span, their increase in health expenditure (as % of GDP) did not contribute significantly to increasing life expectancy and therefore to increasing population health. To some extent, this result is not surprising and can be explained in the light of corruption pressure too which, for some countries, especially those from the former communist bloc, is a higher burden. On the one hand, in most of these countries corruption is part of the health system [11,12]. On the other hand, the lower institutional effectiveness transforms health expenditures into a political instrument for embezzlement, public theft, fraud in government procurements of medical equipment, etc. [2,45]. Thus, even if efforts are made to financially support health systems, resource management is affected by the presence of corruption, which ultimately leads to the maintenance of imbalances in the access of the population to healthcare services.

When population health is measured by child mortality rate, the results displayed in Table 5 suggest that infant mortality is significantly related to corruption, real GDP per capita, unemployment rate, age dependency ratio, government expenditure on education, and degree of urbanization. Nevertheless, similar to the relationship between life expectancy and government health expenditure, chosen as an indicator to measure population access to healthcare services, its impact on child mortality remains insignificant. As previous studies argue [9,33,61,68,69], public health spending is ineffective in lowering the under 5 (child) and infant mortality rate, especially in countries with a high level of corruption or with very ineffective bureaucracy. The other results are overall robust—in signs, magnitude, and significance of coefficients—to the three different estimation specifications and, at the same time, are consistent with previous evidence on developed and developing countries. Therefore, our findings are in line with several studies [8,39,45,57,58] that also reveal a higher level of corruption increases infant mortality rate. According to [58], corruption deepens the increase in infant mortality, especially through income inequalities, which further creates and encourages inequalities in access to education, learning and training opportunities, healthcare, and other resources that should ensure a decent standard of living for the entire population. This point of view is also supported by our study since it offers empirical evidence for the association between infant mortality and other important socio-economic and demographic determinants. In this respect, an increase in GDP per capita, higher government expenditure on education (as % of GDP), and higher urban concentrations contribute significantly to decreasing infant mortality. In addition, the positive response of infant mortality to unemployment rates and age dependency ratio aligns our study to existing literature which documents that a consolidated and stable labor market ensures better living conditions and access to high-quality public services [2] and thus contributes to improving the health of the population, lowering mortality rates, and increasing life expectancy [39].

## 5. Discussion

Our results are on the same wavelength with the perspective provided by New Institutional Economics, via Acemoglu and Robinson, [1] that perceives corruption as an institutional anomaly which will negatively impact on the health of the population. Even though the situation in the EU allows for subsequent positive adjustments, through the improvement of formal policies and rules implemented to support health outcomes, some member countries may be confronted with a delay because of their internal vulnerabilities, reflected in the limited aid provided to the health sector.

Population health is directly related to the level of corruption, which highlights the perspective of businesspeople, citizens, and experts with respect to corruption in the public sector [4]. Consequently, new initiatives undertaken by the EU should focus more on countries from the Central, Eastern and Southern areas, where, apparently, there is an incremental need to strengthen informal institutional conditions through education, civilization, and the mentality of people as to refuse to tolerate or, moreover, to embrace corrupt practices in their everyday life. Such a change of people’s behavior is, in institutional terms, a precondition for better health outcomes. When members of society will refuse the existence and circulation of informal payments and bribes in order to have access to healthcare facilities, the anomaly of corruption will affect less the quality of life in EU and, therefore, the health of the population. For countries that have experienced communism in the past, institutions are extractive and have a higher propensity to use political power as a tool for satisfying the needs of those who are in charge of political institutions [1,15]. Obviously, such inner incremental change is vital, otherwise the formal institutional background provided by EU regulations will not generate the expected positive externalities. Our findings also point out that corruption is not the only issue that must be targeted in order to boost outcomes at the country level in the EU. Good governance must also be provided in terms of improved attention paid to a more efficient management of public funds oriented towards health and education, a consolidated and stable labor market that is less exposed to unemployment and where age dependency is limited, and also to better living conditions wherein the access of people to high quality public services is ensured [2].

The pressure of corruption tends to be higher when the healthcare area is addressed because there is an accentuated insufficiency of public expenditures oriented towards healthcare in most EU countries. Our results reveal that an increase in corruption leads to a decrease in life expectancy at birth and an increase in child mortality rate. These findings can be explained by the fact that in countries with higher levels of corruption, the population’s access to health services is limited and the quality of health services is reduced. This effect of corruption is obviously maintained by different mechanisms such as bribery, diversion of funds, nepotistic appointments in the civil service, lack of access to information on public affairs or government activities, etc. [50]. Even though most EU countries have achieved universal health coverage, resource management is affected by corruption, which ultimately leads to the maintenance of imbalances in people’s access to health services and a rather negative effect on self-rated health [91]. Therefore, people from the most disadvantaged groups tend to have poorer access to health services. For instance, some people may not know or want to use the full range of health services available to them. Furthermore, the quality of healthcare may be worse in more socially deprived areas, and this is a reason why corruption can be perceived as a potentially important source of poor health outcomes [92]. At the same time, some forms of corruption are supported and encouraged by people through informal payments so as to benefit from public healthcare facilities, which in the end will disproportionately affect poor people. In addition, corruption indirectly determines poor performance in medical outcomes and weakens the medical system by encouraging high-quality medical personnel to leave the country and search for better job opportunities abroad. Developing countries from the EU are harshly confronted with the phenomenon of brain drain, an aspect that will further sensitize the labor market with a negative impact in the long run upon economic dynamics [93]. Therefore, when addressing population health, increased attention should be also paid to inclusive formal rules, meaning measures and policies designed to fruitfully explore internal human capital resources for the benefit of society, by protecting longevity and providing people an auspicious labor market where talents can be oriented towards their most productive activities. A fragile labor market with higher rates of unoccupied labor will provide additional pressure on the financing scheme of the public health sector, where most EU countries share the social security health model, meaning that a higher share of the active population will become exposed to poverty and will be left outside the healthcare system. In such conditions, the propensity of patients to use the tool of informal payments to benefit from healthcare facilities will increase. It is a sort of vicious circle that has only one exit road: to build and strengthen the internal regulations, which should be created in order to protect the basic needs of civilians, while sanctioning deviant behaviors and limiting corruption. These are the inclusive ‘rules of the game’ and their building or consolidation process needs time, but they are not impossible to reach [17].

## 6. Conclusions

This paper has investigated the impact of corruption on population health among EU countries from the perspective of New Institutional Economics, according to which the phenomenon of corruption illustrates an anomaly that is more prominent in those countries where political and economic institutions provide auspicious conditions for it. As the existing body of research focused on the nexus between institutions and prosperity has emphasized [1,15,17,18,22,29,94], the ‘rules of the game’, formal or informal, and political or economic, have the power to determine the path of a country towards a positive evolution or, to the contrary, on a negative one.

Corruption has fertile ground to expand precisely in those nations where political and economic institutions are rather extractive and the interests of political leaders prevail. Such context is enabled for countries where their informal institutional background is vitiated by past experiences, such as central planning, and creates propitious circumstances for the appearance of political leaders with a strong affinity for power. Conversely, good governance, or inclusive political and economic institutions, will be developed in countries where the informal ‘rules of the game’ have good origins and are based on sound moral values, transparency, and work ethics, which opens the road towards political leaders with a strong affinity for freedom and wealth. Obviously, such “abuse of entrusted power for private gains” requires a compatible institutional background where the existing political and economic regulations sustain corrupt practices. From such a perspective, the EU provides evidence for both countries with healthy institutions and a strong affinity for prosperity and good quality of life, including positive health outcomes, and countries with a rather poor institutional background that pave the road toward limited economic progress and fragile population health.

The obtained results validated the negative impact of corruption on population health. On the one hand, in those countries where the perceived corruption was high, the life expectancy of the people decreased because of the limited availability of healthcare facilities, medical personnel, or even funding provided to ensure the longevity of people. On the other hand, our results emphasize a positive and significant association between corruption and child mortality, highlighting the same vulnerabilities that sentence to death infants with fragile health conditions. From our point of view, such harsh effects of corruption on overall population health require a dynamic perspective that should be analyzed in time and space. This is an ongoing process which must be understood considering the broader political, social, and economic particularities that define a certain nation. Therefore, institutions can provide an in-depth perspective to the implications of corruption on health outcomes.

We showed that health issues were more noticeable where corruption, as a consequence of ineffective regulations, was high and were less problematic in EU countries where corruption pressure was limited due to inclusive regulations. However, the effectiveness of inclusive or extractive ‘rules of the game’ was also reflected in the general background of each society. Therefore, good governance also ensured economic development and a stable and protected labor market through increased public expenditure oriented towards education and health. In this respect, our analysis pointed out that increasing countries’ economic performance, reducing unemployment, lowering the dependence of young and elderly population on age-working population, allocating a higher percentage of GDP to education expenditures, and an increasing degree of urbanization contributed significantly to improving the health of the population, lowering mortality under 5 years, and increasing total life expectancy at birth. All these achievements could be validated only within a favorable institutional matrix projected on the dimensions of freedom, inclusiveness, and prosperity. The weak relationship between population health—measured both by life expectancy and child mortality—and government expenditure on health (as % of GDP) was not entirely a surprising result in at least two respects. On the one hand, there is the unstable trend in health expenditure in the period after the Great Recession felt by all EU countries, either through weak increases in some states or significant declines in others. On the other hand, corruption, which is much more prominent in public systems, especially in the health sector, of former communist countries has delayed the positive effect of increased health spending on access to quality health services and thus on improving population health.

Our findings confirmed that countries with higher corruption have lower health indicators, i.e., higher mortality and lower life expectancy at the level of EU countries. Moreover, less developed countries have lower health indicators and higher levels of corruption. However, the link between corruption and population health is not just based on differences between less-developed countries and Western European ones. For the latter countries, Transparency International [95] shows that corruption levels have not decreased over the last decade but have either increased or remained at levels close to those of the past. In addition, there are studies showing that corruption increases mortality rates and decreases life expectancy not only in CEE countries, but also in developed countries in Western Europe and North America [51].

In the extension of our analysis, we can draw some policy recommendation to improve population health among EU countries. First, life expectancy should be targeted through incremental increases of educational level and consequently higher expenditure on education, as most developing EU countries have less than 5% of their GDP allocated to education. Higher education creates opportunities and means through which people can improve the economic and social conditions they work and live in. Educated people adopt healthier lifestyles with positive effects on global health. Perceiving things from the perspective of patients, educated adults will value their health status more, while from the viewpoint of the medical labor force, better-qualified human capital will increase the performance, knowledge, and skills of the medical sector. Second, serious improvements in medical care are still required, especially in developing economies in the EU. According to the latest data, improvements concerning the causes of child deaths, prematurity, birth defects, and low birth-weight infants are most often nominated. Consequently, such problems could be targeted by improving the level of income and living conditions, but also through better disease prevention among disadvantaged groups. Better access to health services for low-income groups of people will reduce health inequalities among society members, also having a positive impact on general population health status or even on further economic progress. Poor health is also associated with higher unemployment and poverty; therefore, an overall improvement of health will subsequently influence the other variables. Another important aspect, from our perspective, is the need to reform the access to medical health care, especially for those people with limited financial possibilities, who face barriers such as higher costs of medical care or limited availability of medical facilities in those geographical locations with a low degree of urbanization. From here, other significant problems arise, such as the uneven distribution of physicians and the difficulties in recruiting and retaining these specialists in remote rural regions with sparse populations [54]. Motivating doctors to practice in those regions without worrying for their career, social amenities for their family members, or the working conditions placed at their disposal remain massive challenges for the policy makers from most of the EU’s emerging economies that need to be further addressed. Our endeavor has several limitations given: First, the incomplete availability of the time series for our data considering the countries from the EU. For our sample, due to the small panel size, the analysis by subsamples does not provide robust results and therefore is not included in this study. Another limitation derives from that fact that our analysis has not taken into consideration the current challenge of the COVID-19 pandemic and its massive impact on population health across EU countries, as well as the resilience of the medical sector in the wake of such a shock. Even so, we intend to further explore this new research dimension in our future work, addressing other future directions which are worthy of being considered. First, we intend to apply analysis at a different scale by also taking into consideration specific particularities, considering the health systems from various EU countries, namely Beveridge and Bismarck. Further, we are interested in further investigating if the perspective of New Institutional Economics on the bidirectional relationship between the quality of rules and outcomes remains valid for the corruption/population health nexus by focusing on specific groups of countries from all over the world.

## Figures and Tables

**Table 1:** Variables description and source of data.

Variables	Description	Data Source
Life_expectancy	Life expectancy at birth, total (years)	World Bank
Child_mortality	Mortality rate, under –5 (per 1000 live births)	World Bank
CPI	Corruption Perceptions Index ranks of countries around the world, based on how corrupt their public sectors are perceived to be.	Transparency International
GDP	Gross Domestic Product per capita (current US $)	World Bank
Unempl	Unemployment, total (% of total labor force)	World Bank
Age_depend	Age dependency ratio (% of working-age population), which is the sum of the young population (under age 15) and elderly population (age 65 and over) relative to the working-age population (ages 15 to 64). Data are shown as the number of dependents per 100 working-age population.	World Bank
Educ_expend	General government education expenditure as percentage of GDP	EUROSTAT
Health_expend	General government health expenditure as percentage of GDP	EUROSTAT
Urb	Degree of urbanisation, which represents the urban population as percentange of total population of the country.	EUROSTAT

**Table 2:** Descriptive statistics of the variables used in analysis.

Variables	N	Mean	Std. Dev.	Min.	Max.
Life_expectancy	560	78.2280	3.2411	70.26	83.49
Child_mortality	560	5.4998	2.8663	2.10	21.40
Rank_CPI	560	14.4964	8.0733	1.00	28.00
GDP	560	29,377.89	20,762.59	1621.24	118,823.65
Unempl	560	8.6646	4.3675	2.00	27.50
Age_depend	560	49.2965	4.5994	38.46	61.80
Educ_expend	560	5.1296	0.9402	2.80	7.10
Health_expend	560	5.7888	1.5637	2.18	9.27
Urb	560	72.2296	12.4690	50.75	98.04

Source: Authors calculations.

**Table 3:** Panel unit root tests.

Variables	Level			First Difference		
	Breitung	IPS	Fisher-ADF	Breitung	IPS	Fisher-ADF
Life_expect	0.720	1.868	57.590	−3.864 ***	−9.632 ***	188.81 ***
Mortality	2.621	3.166	69.158	−2.745 ***	−1.859 **	85.192 ***
Rank CPI	−4.351 ***	−1.800 **	72.921 *	-	-	-
GDP	−0.800	0.466	39.438	−12.980 ***	−9.948 ***	195.899 ***
Unempl	−2.789 ***	−1.782 **	75.728 **	-	-	-
Age_Depend	9.031	−1.848 **	113.607 ***	-	-	-
Educ_expend	−3.337 ***	−2.694 ***	86.244 ***	-	-	-
Health_expend	−1.514 *	−1.292 *	70.389 *	−10.205 ***	−7.674 ***	157.295 ***
Urb	0.442	5.835	51.801	−1.184	−6.203 ***	150.082 ***

Notes: *** indicates significance at the 1% level; ** indicates significance at the 5% level; * indicates significance at the 10% level. Variables were abbreviated as follows: Life_expect —life expectancy; Mortality—child mortality (under 5 years); Rank_CPI—rank of Corruption Perception Index; GDP—GDP per capita; Unempl—unemployment rate; Age_depend—age dependency ratio; Educ_expend—general government education expenditure (as % of GDP); Health_expend—general government health expenditure (as % of GDP); Urb—degree of urbanization.

**Table 4:** Estimates of the panel regression models: the impact of corruption on total life expectancy at birth.

Variables	FE Model (1)	RE Model (2)	GMM Model (3)
Constant	0.681 (0.305) ***	−0.501 (0.177) ***	−0.302 (0.330)
Rank CPI	−0.009 (0.004) **	−0.002 (0.002)	−0.002 (0.001) *
DGDP	1.43 × 10^−5^ (7.25 × 10^−6^) ***	1.58 × 10^−5^ (4.23 × 10^−6^) ***	1.41 × 10^−5^ (6.47 × 10^−6^) **
Unempl	0.005 (0.002) **	0.008 (0.003) **	0.002 (0.002)
DAge_depend	−0.014 (0.004) ***	−0.011 (0.003) ***	−0.013 (0.004) ***
Educ_expend	0.059 (0.034) *	0.041 (0.015) ***	0.109 (0.040) ***
DHealth_expend	−0.013 (0.037)	−0.048 (0.042)	−0.013 (0.038)
DUrb	0.146 (0.061) **	0.055 (0.058)	0.151 (0.055) ***
Fisher test (country- and time-specific)	1.876 **	-	-
Hausman (country- and time-specific)	-	17.408 **	-
$\mathrm{Adjusted}\;R^{2}$ (country- and time-specific)	0.315 ***	0.073 ***	0.299 ***

Notes: Standard errors in parentheses. *** indicates significance at the 1% level; ** indicates significance at the 5% level; * indicates significance at the 10% level. Variables were abbreviated as follows: Rank_CPI—rank of Corruption Perception Index; DGDP—first difference of GDP per capita; Unempl—unemployment rate; DAge_depend—first difference of age dependency ratio; Educ_expend—general government education expenditure (as % of GDP); DHealth_expend—first difference of general government health expenditure (as % of GDP); DUrb—first difference of degree of urbanization.

**Table 5:** Estimates of the panel regression models: the impact of corruption on child mortality (under 5 years).

Variables	FE Model (4)	RE Model (5)	GMM Model (6)
Constant	−0.7759 (0.1285) ***	−0.7372 (0.1693) ***	−0.6587 (0.1395) ***
Rank CPI	0.0076 (0.0014) ***	0.0103 (0.0021) ***	0.0095 (0.0015) ***
DGDP	−3.95 × 10^−6^ (1.21 × 10^−6^) ***	−6.15 × 10^−6^ (1.43 × 10^−6^) ***	−4.09 × 10^−6^ (1.26 × 10^−6^) ***
Unempl	0.0034 (0.0015) **	0.0051 (0.0021) **	0.0035 (0.0016) **
DAge_depend	0.0175 (0.0014) ***	0.0195 (0.0022) ***	0.0016 (0.0013) ***
Educ_expend	−0.0407 (0.0147) ***	−0.0618 (0.0135) ***	−0.0446 (0.0174) **
DHealth_expend	−0.0104 (0.0097)	−0.0191 (0.0235)	−0.0131 (0.0095)
DUrb	−0.0544 (0.0162) ***	−0.0582 (0.0347) *	−0.0531 (0.0157) ***
Fisher test (country- and time-specific)	19.9862 ***	-	-
Hausman (country- and time-specific)	-	59.132 ***	-
$\mathrm{Adjusted}\;R^{2}$ (country- and time-specific)	0.6544 ***	0.2729 ***	0.6750 ***

Notes: Standard errors in parentheses. *** indicates significance at the 1% level; ** indicates significance at the 5% level; * indicates significance at the 10% level. Variables were abbreviated as follows: Rank_CPI—rank of Corruption Perception Index; DGDP—first difference of GDP per capita; Unempl—unemployment rate; DAge_depend—first difference of age dependency ratio; Educ_expend—general government education expenditure (as % of GDP); DHealth_expend—first difference of general government health expenditure (as % of GDP); DUrb—first difference of degree of urbanization.

## Data Availability

Publicly available datasets were analyzed in this study. More details regarding the series used in the study are displayed in Table 1.

## Appendix A

List of countries included in our sample:

Austria, Belgium, Bulgaria, Croatia, Cyprus, Czech Republic, Denmark, Estonia, Finland, France, Germany, Greece, Hungary, Ireland, Italy, Latvia, Lithuania, Luxembourg, Malta, Netherlands, Poland, Portugal, Romania, Slovak Republic, Slovenia, Spain, Sweden and United Kingdom.
